# Riboflavin- and Dextran-Producing *Weissella confusa* FS54 B2: Characterization and Testing for Development of Fermented Plant-Based Beverages

**DOI:** 10.3390/foods13244112

**Published:** 2024-12-19

**Authors:** Malek Lahmar, Norhane Besrour-Aouam, Annel M. Hernández-Alcántara, Iñaki Diez-Ozaeta, Imene Fhoula, Paloma López, Mari Luz Mohedano, Hadda-Imene Ouzari

**Affiliations:** 1 Centro de Investigaciones Biológicas Margarita Salas (CIB-CSIC), 28040 Madrid, Spain; lahmar.malek17@gmail.com (M.L.); annel@cib.csic.es (A.M.H.-A.); i.diez08@hotmail.com (I.D.-O.); plg@cib.csic.es (P.L.); 2 Microorganisms and Active Biomolecules Laboratory (LR03ES03), Faculty of Sciences of Tunis, University of Tunis El Manar, Tunis 2092, Tunisia; bnorhane@hotmail.fr (N.B.-A.); imene.fhoula@fst.utm.tn (I.F.); imene.ouzari@fst.utm.tn (H.-I.O.)

**Keywords:** lactic acid bacteria, *Weissella confusa*, riboflavin, FMN riboswitch, plant-based beverages, prebiotic oligosaccharides, dextran

## Abstract

The use of lactic acid bacteria for developing functional foods is increasing for their ability to synthesize beneficial metabolites such as vitamin B (riboflavin, RF) and postbiotic compounds. Here, the spontaneous mutant FS54 B2 was isolated by treatment of the dextran-producing *Weissella confusa* FS54 strain with roseoflavin. FS54 B2 overproduced RF (4.9 mg/L) in synthetic medium. The FMN riboswitch is responsible for the regulation of RF biosynthesis, and sequencing of the coding DNA revealed that FS54 B2 carries the G131U mutation. FS54 B2 retained the capacity of FS54 to synthesize high levels of dextran (3.8 g/L) in synthetic medium. The fermentation capacities of the two *Weissella* strains was tested in commercial oat-, soy- and rice-based beverages. The best substrate for FS54 B2 was the oat-based drink, in which, after fermentation, the following were detected: RF (2.4 mg/L), dextran (5.3 mg/L), potential prebiotics (oligosaccharides (panose (5.1 g/L), isomaltose (753 mg/L) and isomaltotriose (454 mg/L)) and the antioxidant mannitol (16.3 g/L). pH-lowering ability and cell viability after one month of storage period were confirmed. As far as we know, this is the first time that an RF-overproducing *W. confusa* strain has been isolated, characterized and tested for its potential use in the development of functional beverages.

## 1. Introduction

The utilization of lactic acid bacteria (LAB) is increasing due to several factors. Aside from their fermentative abilities, many LAB strains possess probiotic properties, and some of them are deemed food-grade microorganisms, labeled as safe for both humans and animals with the Qualified Presumption of Safety (QPS) provided by the European Food Safety Administration (EFSA). Furthermore, they are capable of synthesizing postbiotic compounds. Thus, LAB provide significant benefits to their hosts, allowing them to be frequently employed as probiotics [1].

LAB are a diverse group of Gram-positive bacteria with low G+C content in their DNA and currently include species belonging to 64 genera [2]. Among them, LAB belonging to the genus *Weissella* have emerged in recent decades as very promising for the food industry due to their probiotic and biotechnological potential. *Weissella* spp. are found in a wide range of ecological niches, including plants, vegetables, soil and water. Moreover, they are present in a variety of fermented food (such as sausages, fermented fish, pickles, fermented coconut, fermented seafood and fermented rice grains), where they can assume a probiotic role [3]. Thus, *Weissella* species, particularly *Weisella cibaria* and *Weisella confusa*, have been identified as starter cultures for food fermentation processes to enhance food quality and functionality, as well as potential probiotics to promote health [4]. Although they have not yet attained QPS status and there are some controversial opinions, in silico analysis of the genome of 46 *W. confusa* strains, testing for undesirable properties, such as antibiotic resistance or hemolytic activity, as well as bibliographic surveys support the safety of this species for food usage [5].

In addition, *Weissella* strains are widely documented for their capability to synthesize a diverse range of exopolysaccharides (EPSs), which have potential industrial applications and bio-functional properties [6]. The production of EPSs by starter cultures is commonly favored by the dairy industry and, in the case of sourdough fermentation, improves the texture and storage life of bread [7]. In particular, *Weissella* strains produce dextran at high levels [8], and the in situ production of dextran by *W. confusa* CK15 strain has been shown to improve rheological properties of chickpea sourdough [9].

Dextrans are α-glucan polymers characterized by major chains comprising glucose molecules linked by α-(1–6) bonds, with branches extending from α-(1–2), α-(1–3), and α-(1–4) linkages [10]. These compounds are synthesized by diverse LAB, including lactobacilli [11] and species belonging to the genera *Leuconostoc* [12], *Streptococcus* [13] and *Weissella* [14,15,16]. Dextrans can act as hydrocolloid agents, increasing water retention and viscosity of food matrices without affecting their flavor. Thus, dextrans provide organoleptic, sensory and rheological properties to various products. Consequently, the dextrans are widely used as food additives, particularly in the baking industry and in the production of confectionary, such as ice creams and milkshakes, to enhance palatability [16,17]. Additionally, the dextrans are GRAS, and as postbiotics, they may provide human health benefits, such as antioxidative, immunomodulatory, antimicrobial, anti-tumor or anti-inflammatory activities [18,19].

Vitamin B_2_, commonly known as riboflavin (RF), is a water-soluble vitamin essential for the metabolism of all living organisms. However, only plants and microorganisms have the capacity to synthesize it [20]. RF is essential for several biochemical processes because it serves as a precursor for crucial coenzymes, namely flavin adenine dinucleotide (FAD) and flavin mononucleotide (FMN). These flavocoenzymes play a pivotal role in the redox reactions that occur in various organisms. They actively participate in the metabolism of carbohydrates, lipids, ketone bodies and proteins, which serve as the primary sources of energy for living organisms [20].

Humans rely on obtaining most vitamins exogenously through a balanced diet, as their synthesis is limited to the microbiota in the body [21,22]. RF can be found in various food sources such as milk, rice, meat, eggs, nuts, and vegetables. The recommended dietary allowances (RDAs) for RF intake differ between human and animal nutrition, typically ranging from 0.4 to 0.6 mg/day for humans and 0 to 17.5 mg/kg for animals [23,24,25]. According to the European Food Information Council, the recommended daily amount for riboflavin is 1.6 mg per day [25]. RF deficiency, known as ariboflavinosis, can lead to health issues such as liver and skin damage, neurological changes, and impaired glucose metabolism. Additionally, riboflavin has been implicated in the prevention of diseases such as cancer, migraines, anemia, hypertension, and in reducing oxidative stress [26,27].

Despite its presence in a wide variety of foods, RF deficiency remains prevalent worldwide in both developed and developing countries due to inadequate dietary intake [28]. This deficiency has sparked interest in riboflavin-biofortified foods utilizing RF-overproducing LAB, which are seen as a natural, consumer-friendly, and sustainable alternative to chemically synthesized RF [28].

However, most LAB produce low levels of RF, and the introduction of genetically modified microorganisms that overproduce RF still faces significant challenges within the industry, primarily stemming from consumer and regulatory concerns regarding genetic engineering [29].

In this context, biotechnological methods for the detection of food-grade LAB strains capable of overproducing vitamin B_2_ were explored for their potential application in the development of functional foods. RF synthesis involves four proteins (RibG, RibB, RibA and RibH), encoded by genes within the *rib* operon. The expression of this operon is regulated by attenuation through the binding of FMN to the riboswitch located in the 5′-untranslated region of the *rib* mRNA. This riboswitch contains the acceptor FMN-binding aptamer, which also binds roseoflavin, a toxic analog of riboflavin. Thus, exposure of RF-producing LAB strains to roseoflavin has proven to be a reliable method for obtaining RF-overproducing strains carrying mutations at the FMN riboswitch. This technique has successfully led to the selection of spontaneous roseoflavin-resistant RF-overproducing LAB strains, including those belonging to the species *Lactococcus lactis* [30], *Lactiplantibacillus plantarum* [31,32,33], *Limosilactobacillus fermentum* [34], *Leuconostoc mesenteroides* [31] and *W. cibaria* [35,36].

It is important to emphasize that these RF-resistant strains are spontaneous, non-genetically modified organisms, making them potentially suitable for the production of vitamin B_2_-fortified foods. Recent studies have reported RF fortification using RF-producing LAB strains isolated from various food sources, including milk, soy milk, whey, kefir-type cereal drinks, and pseudocereals [37,38,39]. Bread and pasta have been developed as functional foods, owing to their inherent properties that facilitate vitamin fortification [34,37].

In this context, LAB have shown promising results for in situ biofortification of various foods, using strains from different species such as *L. plantarum* [33], *L. fermentum* [34], *Limosilactobacillus reuteri* [39], or *W. cibaria* [35,40]. The ability to increase RF levels in food matrices lacking or containing low concentrations of this vitamin is of significant interest to the food and health sectors. This is particularly relevant for plant-based products, which are becoming increasingly popular as a healthy alternative to dairy products and offer a potential avenue for the development of potential functional beverages [41].

In this study, with the aim of broadening the spectrum of LAB species for the development of plant-based functional beverages, we selected and isolated the spontaneous mutant strains FS54 B2 from roseoflavin-treated cultures of the dextran- and RF-producing *W. confusa* FS54 strain isolated in Tunisia [42]. After isolation, we confirmed that FS54 overproduces RF and retains the dextran synthesis capability of the parental strain. We determined the sequence of its *rib* FMN riboswitch and predicted the consequences of its point mutation on the folding of the riboswitch aptamer. Furthermore, to explore the potential applications of *W. confusa* FS54 B2 in the development of new functional beverages, we compared the fermentation capability of FS54 B2 and FS54 in three commercial plant-based beverages, analyzing their RF and dextran production and their sugar metabolism.

## 2. Materials and Methods

### 2.1. Bacterial Strains and Growth Conditions

Two *W. confusa* strains were used in this work: FS54 B2 isolated in this study and FS54 isolated from the rhizosphere of a Tunisian olive tree [42]. The bacteria were grown at 30 °C and propagated routinely in liquid Man, Rogosa and Sharpe rich medium without dextrose (Condalab, Madrid, Spain) supplemented with either 2% sucrose (MRSS) or 2% glucose (MRSG). The Riboflavin Assay Medium (Difco, RAM, Forn El Chebbak, Lebanon) was used for bacterial growth, when the production of RF and dextran by the *W. confusa* strains were investigated or when the FS54 was subjected to treatment with roseoflavin. RAM, a defined medium lacking RF, was prepared by components and supplemented with either 2% glucose (RAMG) or 2% sucrose (RAMS).

The growth rate (μ) per hour and the doubling time (Dt) of the strains were determined as previously described [35].

### 2.2. Isolation of the FS54 B2 Strain

**Figure 1:**
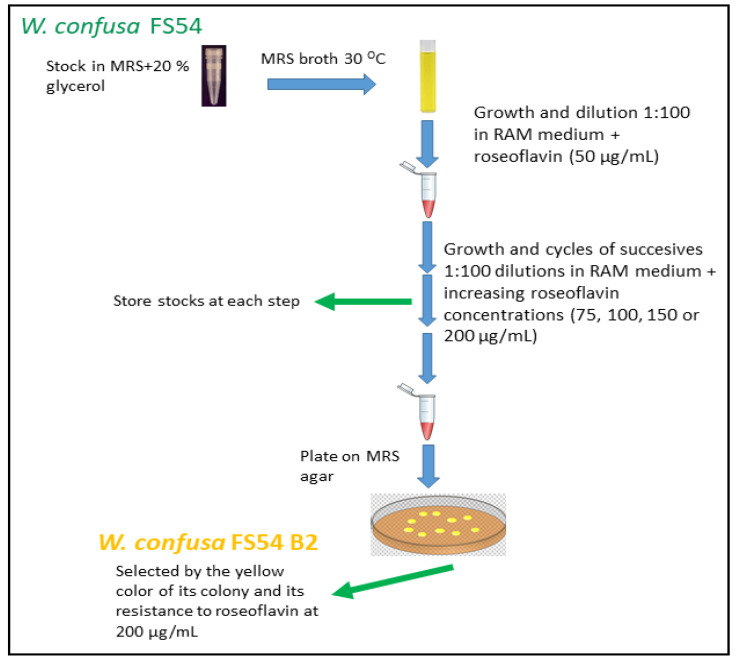
Schematic representation of the methodology followed for the selection and isolation of the *W. confusa* FS54 B2. After treating *W. confusa* FS54 with increasing concentrations of roseoflavin, the FS54 B2 strain was selected and isolated based on the yellow color in MRS.

To isolate the FS54 B2, the protocol showed in Figure 1 was followed. The *W. confusa* FS54 strain was initially grown in MRS at 30 °C until it reached an optical density at 600 nm (OD_600nm_) of 1.5. It was then diluted 1:100 in RAMG medium containing 10 µg/mL of roseoflavin and cultured at 30 °C for approximately 24 h. The cells were then transferred to RAMG medium without roseoflavin. Successive 1:100 dilutions of FS54 were performed in RAMG supplemented with increasing concentrations of roseoflavin (50 µg/mL, 75 µg/mL, 100 µg/mL, 150 µg/mL, and 200 µg/mL). After exposure to 200 µg/mL, a portion of the culture was diluted in phosphate-buffered saline (PBS, 137 mM NaCl, 2.7 mM KCl, 4.3 mM Na_2_HPO, and 1.47 mM KH_2_PO) with pH 7.0, plated on MRS agar, and incubated for 48 h. Finally, a clone identified by the predominant yellowish appearance of its colony was selected, isolated, designated as FS54 B2 strain base on its potential RF-overproducing phenotype, and stored at −80 °C in MRSG supplemented with 20% glycerol.

### 2.3. Analysis of RF and Dextran Production by FS54 and FS54 B2 Strains

For quantitative assessment of RF and dextran, bacteria were grown in 4 mL of MRSG or MRSS at 30 °C until an optical density at 600 nm (OD_600nm_) of 1.0 was reached. The pre-cultures were then used to inoculate 40 mL of RAMG or RAMS at an initial OD_600nm_ of 0.1, followed by further incubation for 16 h at 30 °C. Then, samples of the cultures were used to determine the concentration of RF and dextran as follows.

RF was analyzed directly in samples of cultures and of culture supernatants after removal of the bacteria by centrifugation (at 9700× *g*, 10 min, 4 °C). The bacteria were sedimented by centrifugation, and the supernatants were used to determine their RF fluorescence as previously described [32]. To this end, 200 μL aliquots of each sample in triplicate were dispensed in a 96-well polystyrene optical bottom plate (Thermo Fisher Scientific, Rochester, NY, USA). Finally, the OD_600nm_ and RF fluorescence were detected using a Varioskan Flask System (Thermo Fisher Scientific, Waltham, MA, USA). After measurement of the RF fluorescence, the concentration of the vitamin in the samples was calculated using an RF dissolved in the RAM calibration curve (Supplementary Figure S1A) as previously described [35].

To determine dextran production, bacteria from the above cultures grown in RAMS were sedimented by centrifugation at 7300× *g* for 1 h at 4 °C. The extracellular dextran was recovered from the culture supernatant by precipitation with cold absolute ethanol (*v*/*v*) followed by storage for 15 h at 4 °C in a Liebherr refrigerator (model UGK 6450 Index 20A /001, Bulle, Switzerland). The precipitated dextran was then sedimented by centrifugation at 7300× *g* for 1 h, air-dried, and dissolved in ultrapure water. Subsequently, the dextran preparations were frozen at −80 °C, lyophilized until dry using a VirTis lyophilizer Model 4KBTZL (SP Scientific Products, Ipswish, UK), and their concentration was determined by the phenol-sulfuric acid method as previously described [11].

For detection of RF produced during bacterial growth, the following protocol was used. Overnight cultures grown in MRSS at 30 °C were sedimented by centrifugation at 9300× *g* for 10 min. Afterwards, the LAB were resuspended in RAMS medium and incubated at 30 °C until reaching an OD_600nm_ of 1.0. Afterwards, the cultures were diluted and used to inoculate fresh RAMS medium. Then, 200 μL aliquots of the cultures were dispensed in triplicate in the 96-well polystyrene optical bottom plate. Finally, the OD_600nm_ and the RF fluorescence were monitored every 30 min, at 30 °C until the cultures reached the late stationary phase using the plate reader as described above.

### 2.4. Detection of Dextranase Activity

The FS54 and FS54 B2 strains were grown in RAMG to an OD_600nm_ of 1.0. Then, 10 μL containing 5 × 10^8^ CFUs of each culture was spotted on MRSG-agar plates supplemented with 0.4% dextran blue as previously described [16] and incubated at 30 °C for 30 days. It has to be stated that dextran blue is a soluble polymer compound composed of dextran from *Leuconostoc* spp. covalently linked to the dye cibacron blue, and its addition confers to the MRSG-agar plates a blue color. Thus, when a strain hydrolyzes the dextran blue during growth, a colorless halo appears around the spotted bacteria.

### 2.5. Genomic DNA Extraction, PCR Amplification and DNA Sequencing of the FMN Riboswitches

The extraction of genomic DNA (gDNA) from LAB strains was performed with the Wizard Genomic DNA Purification Kit (Promega, Madison, WI, USA) following the manufacturer’s instructions, with modifications in three steps: (i) The lysis process was enhanced by adding lysozyme (30 mg/mL) and mutanolysin (25 U); (ii) DNA precipitation was performed using isopropanol supplemented with Pellet Paint Coprecipitant NF (Merck, Darmstadt, Germany); and (iii) Instead of the standard washing procedure, residual salts were removed by capillary action before drying the pellet with a vacuum pump for 20 min. The resulting gDNA was then resuspended in 10 mM Tris-HCl (pH 8.0). DNA integrity was then verified via electrophoresis on a 0.8% agarose gel in Tris-Acetate-EDTA buffer (Sigma-Aldrich, Darmstadt, Germany) containing GelRed (Biotium, Fremont, CA, USA). This gDNA served as a template for PCR amplification, following the recombinant Taq DNA polymerase protocol (Thermo Fisher Scientific, Waltham, MA, USA) in a final reaction volume of 50 µL. The primers used were ForRibo (5′-AACACATCAACGGCAATCCC-3′) and ReverseRibo (5′-ACATGGTCTTGCTGGGATCA-3′). The reaction product was a 435 bp fragment including the FMN-riboswitch regulatory region of the *rib* operon. PCR conditions were as follows: preheat at 94 °C for 3 min, followed by 25 PCR cycles of denaturation at 95 °C for 45 s, annealing at 58 °C for 30 s, extension at 72 °C for 90 s, and final extension for 10 min. Successful amplification was confirmed by analyzing the amplicons in a 0.8% agarose gel and capturing images using a Gel Doc 2000 Bio-Rad gel documentation system (Bio-Rad, Hercules, CA, USA), with subsequent analysis using the Quantity One 4.5.2 Bio-Rad software. PCR products underwent purification using the QIAquick PCR Purification Kit (Qiagen, Venlo, The Netherlands) and then subjected to automated sequencing via the dideoxynucleotide method by Secugen (Madrid, Spain).

The obtained sequences were analyzed using Chromas 2.6.6 (Technelysium Pty. Ltd., Brisbane, Australia) and DNASTAR 17 (Lasergene, Madison, WI, USA) software. Comparison of the sequences from the mutant and the wild-type strains was conducted using the BLASTN program [43]. Additionally, secondary structure predictions of the FMN riboswitch aptamers were generated using the RNAfold web server (The ViennaRNA Web Services, version 2.4.18) and VARNA 3.9 software [44].

### 2.6. Fermentations of Plant-Based Beverages

The FS54 and FS54 B2 strains were incorporated into three commercially available plant-based beverages: oat (Alitey oat beverage), soy (Carrefour Soy beverage), and rice (Yosoy rice-based drink).

Overnight cultures of the strains, grown in MRSG and MRSS media, underwent centrifugation at 9300× *g* for 10 min. The bacteria present in the pellet were then resuspended in the corresponding fresh medium (MRSG or MRSS) to obtain an OD_600nm_ of 0.1. Subsequently, cultures were grown to an OD_600nm_ of 1 (approximately 5 × 10^8^ CFU/mL), centrifuged once more, and re-suspended in the respective plant-based beverage to achieve an initial cell density of around 5 × 10^7^ CFU/mL. All beverages were supplemented with 5% sucrose, and fermentations were conducted for 48 h at 30 °C. Samples were collected at 0 h and after 2 days for RF and dextran quantification, pH measurements, and cell viability determination by plating, detecting the colony forming units per mL (CFU/mL). The pH was measured using a Crison pH/mV-meter 501. Following the 2-day fermentation, the beverages were stored under refrigeration at 4 °C for 30 days. Samples were taken at 0 h as well as at 48 h post-fermentation and then every 8 days throughout the storage period. All experiments were performed in duplicate. Samples underwent analysis for the RF content, the pH and the cell viability.

### 2.7. Analysis of RF Present in the Beverages

Prior to RF analysis, a slight pre-treatment of the beverage samples was conducted. Non-fermented control beverages underwent chemical coagulation by addition of 1 M HCl until a pH of 4.0 was reached. Subsequently, both non-fermented and fermented samples were centrifuged at 1000× *g* for 10 min. The resulting supernatants were collected, filtered through a 0.22 µm filter and stored until further analysis. Then, RF quantification via fluorescence detection using the microplate reader was performed as detailed in Section 2.3. RF concentration was then determined using calibration curves of RF dissolved in the corresponding non-fermented beverage (Supplementary Figure S1B–D).

### 2.8. Analysis of Sugar Metabolic Fluxes

Quantification of the sugars and metabolites present in the beverages after 48 h of fermentation was performed as previously described [45]. The filtered supernatants, prepared as indicated in 2.7, were analyzed by gas chromatography-mass spectrometry (GC-MS) using a gas chromatograph 7890A (Agilent, Palo Alto, CA, USA) equipped with a Flame Ionization Detector, Thermal Conductivity Detector, autosampler (150 vials) and Headspace Sampler 7697A (Agilent). Myo-inositol was used as an internal standard and the concentration 4 GC-ChemStation Rev. E.02.00 software from Agilent was used (Palo Alto, CA, USA).

### 2.9. Analysis of Dextran Present in the Beverages

Determination of the soluble dextran present in the beverages after 48 h of fermentation was performed by modification of the method developed by us [45], based on dextran hydrolysis [46] as follows. Fermented drinks were centrifuged at 1000× *g* for 10 min. The resulting supernatants were collected, and samples of 150 µL were heated at 99 °C for 15 min. Then, they were cooled down at room temperature for 3 min, and they were treated with a dextranase solution (15 µL) containing 18 mg of *Chaetomium erraticum* dextranase (Sigma-Aldrich, Darmstadt, Germany) for 17 h at 30 °C to convert dextran into isomaltose. Afterwards, the treated samples were centrifuged at 12,000× *g* for 10 min, and the resulting supernatants were stored at −20 °C until further analysis by GC-MS as detailed in Section 2.8.

### 2.10. Statistical Analysis

All measurements were analyzed by one-way ANOVA. A *p* value of ≤0.05 was considered significant. When ANOVA tests were significant, mean pairwise comparisons were computed with Tukey’s test (α = 0.05). All analyses were performed with the R software version 4.3.1 (R Core Team, 2023) [47].

## 3. Results

### 3.1. Isolation and Initial Testing of the W. confusa FS54 B2 Strain

Thus far, no *W. confusa* strain capable of producing dextran and overproducing RF has been described. Therefore, since we had previously characterized the Tunisian strain FS54 as a high-level dextran producer [16], we set out to select and isolate a mutant of this strain able to overproduce RF. To this end, the FS54 strain was treated with increasing concentrations of roseoflavin according to the protocol described in Figure 1. A strain that was resistant to the RF homologue at a concentration of 200 μg/mL was obtained.

**Figure 2:**
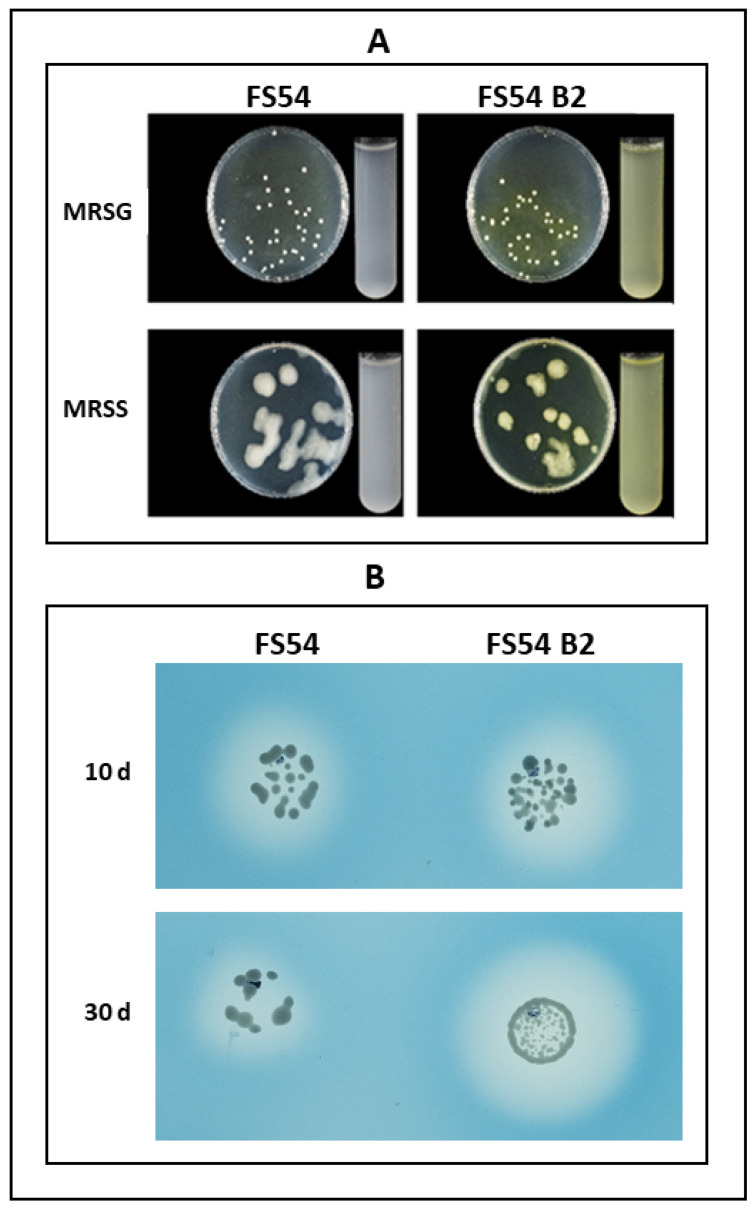
Macroscopic detection of *W. confusa* FS54 and FS54 B2 characteristics. (**A**). Detection of dextran and RF production is depicted. The photographs show bacteria grown for 48 h at 30 °C on either MRSG- or MRSS-agar solid medium and in RAMG or RAMS liquid medium. (**B**). Detection of dextranase activity in MRSG-agar–dextran-blue medium is shown. The photographs show colorless halos of dextran degradation surrounding the bacteria grown at 30 °C for 10 days or 30 days.

After isolation, this selected spontaneous mutant was designated FS54 B2, and its ability to produce RF and dextran in liquid and solid media was ascertained by comparison with the behavior of the parental FS54 strain under the same conditions (Figure 2A). FS54 B2 grown on both MRSS and MRSG solid or liquid media showed a yellow color, in contrast to FS54, which exhibited a white color phenotype.

The synthesis of dextran by the dextransucrases (Dsr) requires sucrose as substrate. Therefore, as expected, FS54 B2, like its parental strain, formed large distinctive mucous colonies due to the production of dextran when grown on MRSS-agar but not on MRSG-agar (Figure 2A).

In addition, we previously detected dextranase activity catalyzed by FS54 [16]; therefore, it was evaluated whether FS54 B2 retained this activity by spotting and further analysis on MRSG-agar plates supplemented with 0.4% dextran blue.

As shown in Figure 2B, after 10 days of incubation, colorless halos due to dextran blue degradation were detected on plates inoculated with either FS54 or FS54 B2. Furthermore, after 30 days of incubation, the mutant produced a larger halo than the parental strain. Therefore, these results indicate that *W. confusa* FS54 B2 is an RF overproducer and a dextran producer and has dextranase activity.

### 3.2. Analysis of the RF and Dextran Production of the W. confusa FS54 B2 and FS54 Strains in Synthetic Growth Media

This study evaluated the bacterial growth as well as the RF and the dextran production of FS54 B2 compared to FS54.

We have previously demonstrated that bacteria belonging to *Leuconostoc* species and *W. cibaria* strains were able to produce dextran when grown in the RAM defined medium without RF, which lacks polysaccharides, when it contained sucrose, the substrate for the synthesis of dextran by the extracellular dextransucrases Dsr [15,36]. Therefore, we decided to use the RAM medium containing either sucrose or glucose to perform the comparative analysis of the *W. confusa* strains, expecting a similar good dextran production by them in RAMS and a differential RF synthesis among them in both RAMS and RAMG. Therefore, the *W. confusa* strains were grown for 16 h at 30 °C in RAMS or RAMG, and their final OD_600nm_ was measured. Their RF production was also determined by measuring the total fluorescence of the cultures and the fluorescence present in the culture supernatants. Moreover, the dextran present in the culture supernatants was partially purified by ethanol precipitation and quantified. The results obtained are presented in Table 1. Both bacteria showed good growth in the two media tested, with a final OD_600nm_ ranging from 4.3 to 3.1. Regarding dextran production, as anticipated, no production was observed by either *W. confusa* strain in RAMG. In RAMS, both strains synthesized the same high levels of dextran (3.8 g/L). Thus, these results confirmed that FS54 B2 retained the capacity of FS54 for dextran production.

Concerning the RF production, both the parental and mutant strains synthesized higher amounts of RF in RAMS medium compared to those detected in RAMG. The FS54 strain consistently exhibited a low RF production (0.3 mg/L and 0.2 mg/L in RAMS and RAMG, respectively), a result in accordance with previous observations for *W. cibaria* wild-type strains [35,36]. By contrast, FS54 B2 produced statistically significantly high levels of RF (4.9 mg/L and 3.9 mg/L in RAMS and in RAMG, respectively), representing concentrations 16- and 19-fold higher than that produced by FS54 in the same conditions. In addition, in the case of FS54 B2 cultures, more than 95% of the total RF produced in RAMS and over 80% of that which was produced in RAMG were detected in the corresponding culture supernatants, indicating that most of the vitamin B_2_ synthesized by the bacteria was secreted. However, the FS54 strain released a lower proportion of vitamin B_2_ into the supernatant, 66% and 50% in RAMS and RAMG, respectively. Thus, the above results confirmed that *W. confusa* FS54 B2 is an RF-overproducing strain.

**Figure 3:**
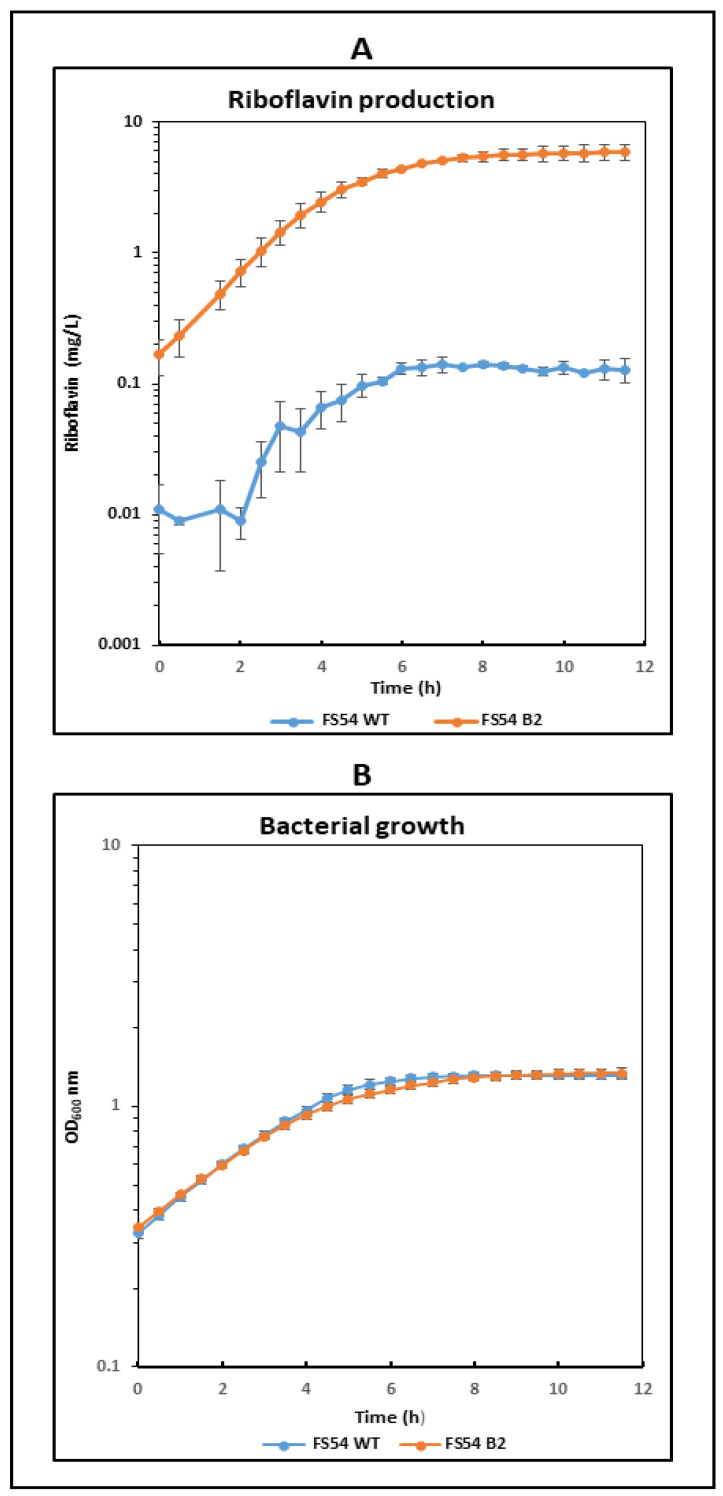
Comparative real-time analysis of RF production and growth of *W. confusa* FS54 and FS54 B2 strains. LAB were grown in RAMS at 30 °C in microtiter plates and analyzed in a Varioskan equipment. (**A**). The levels of RF estimated from the fluorescence measurements of the cultures are depicted. (**B**). Bacterial growth estimated by detection of the OD_600nm_ of the culture is shown.

To obtain further information on the RF production, during the growth of the *W. confusa* strains in RAMS, the fluorescence of the culture due to the vitamin B_2_ and the OD_600nm_ was continuously monitored in real time during growth. Furthermore, the RF concentrations during growth were inferred from the fluorescence monitoring. The evolution of the RF levels and of the OD_600nm_ is presented in Figure 3.

In the FS54 B2 cultures, the production of RF (Figure 3A) and the increase in OD_600nm_ (Figure 3B) were observed during all the detected exponential growth phase (Figure 3B), as it was previously observed for RF-overproducing *W. cibaria* mutants [15,36], and as expected for RF synthesis depending on the biomass. By contrast, during the exponential growth phase of FS54, an initial delay of 2 h was monitored prior to starting the RF production (Figure 3A), although no latent period was observed in the OD_600nm_ measurements (Figure 3B). We previously observed this behavior in *W. cibaria* BAL3C-5, BAL3C-7 and BAL3C-22 wild-type strains, showing a latent period for initiation of RF production of approximately 1 h, when they were grown in RAMS, and up to 2 h when the growth medium was supplemented with RF or FMN [15,36]. Thus, the general behavior of the *Weissella* wild-type strains could be explained by a repression of the *rib* operon expression due to initial intracellular levels of RF in the bacteria that need to be consumed prior to the start of vitamin synthesis.

In addition, at the stationary phase in the FS54 B2 cultures, up to 6 mg/L of RF was detected, a 42-fold higher level than that in the FS54 cultures, although both strains reached similar final OD_600nm_.

Moreover, only a slightly faster growth of the mutant was detected, as FS54 B2 had a μ of 0.25 ± 0.03 per hour and a D_t_ of 2.80 ± 0.40 h, whereas FS54 showed a μ of 0.24 ± 0.06 per hour and a D_t_ of 2.85 ± 0.44 h. However, FS54 B2 started to enter in the stationary phase after 4 h of incubation and FS54 reached this phase after 6 h of growth.

Finally, the mutant showed the highest rate of RF production (0.61 ± 0.01 mg/h/L of culture) during the first 4 h of incubation, whereas the wild-type strain only reached a rate of 0.36 ± 0.12 in the 2–6 h range of incubation.

Thus, the overall results confirmed that the FS54 B2 strain was a very good RF overproducer under conditions where high levels of dextran could be produced and indicated that the synthesis of the vitamin B_2_ by the mutant was dysregulated.

### 3.3. Genetic Analysis of the RF-Overproducing FS54 B2 Strain

Roseoflavin-resistant mutants of LAB typically carry mutations that decrease or impair the inhibitory regulatory function of the FMN riboswitch of the *rib* operon, allowing the bacteria to produce RF even when FMN or roseoflavin is present in the cell. Therefore, to identify and characterize the potential mutation(s) responsible for the RF-overproducing phenotype, the DNA sequence of the *rib* FMN riboswitch for both the FS54 B2 and its parental FS54 strains was determined. Only one nucleotide change from G to T was observed in the DNA of the mutant strain. This substitution was located at position 131 of the coding sequence of the riboswitch aptamer involved in FMN binding.

**Figure 4:**
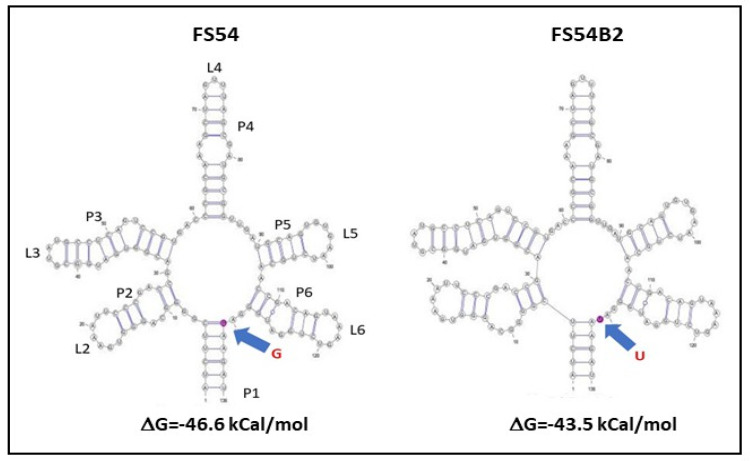
Predicted folding of the FMN-riboswitch aptamer of *W. confusa* FS54 and FS54 B2 strains. The folding of the aptamer and calculations of the -ΔG of its formation are depicted and obtained by using the RNAfold web server (The ViennaRNA Web Services, version 2.4.18) and VARNA 3.9 software. The position of the G131U mutation in the FS54 B2 and the wild-type aptamer are indicated with blue arrows.

Prediction of the folding of the FS54 B2 and FS54 aptamers using RNAfold and VARNA software indicated the presence of five stem-loop structures (P2/L2-P6/L6) and a terminal P1 structure (Figure 4), characteristics of the FMN binding site of bacterial riboswitches [48,49]. Moreover, the in silico analysis revealed that the substitution (G131U in the RNA) induced a change in the RNA folding, since the ribonucleotide G131 was located in the P1 stem of the wild-type aptamer, whereas the U131 was included in the region between P6 and P1 in the mutant aptamer. As a result, the mutation decreased the Gibbs free energy (ΔG) of the aptamer formation from −46.6 kCal/mol in FS54 to −43.5 kCal/mol in FS54 B2.

Therefore, this mutation could play a role in the deregulation of *rib* operon expression, by decreasing the frequency of aptamer formation and consequently the FMN binding to the aptamer, resulting in the RF-overproducing phenotype of *W. confusa* FS54 B2.

### 3.4. Fermentations of Plant-Based Beverages with the W. confusa FS54 B2 and FS54 Strains

**Figure 5:**
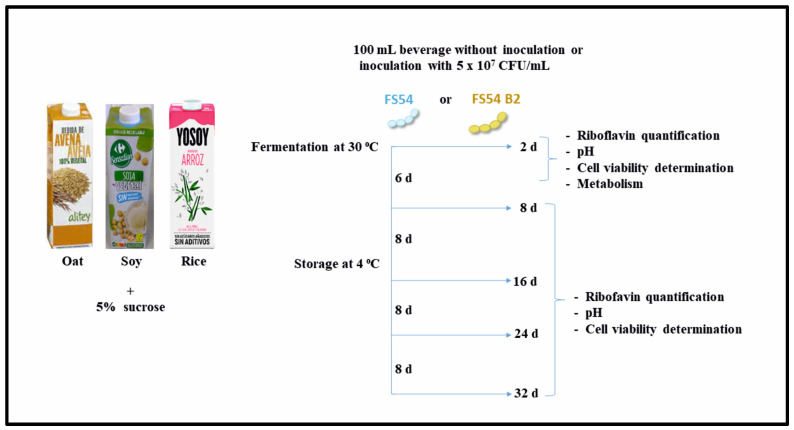
Diagram outlining the methodology for fermentation of plant-based beverages with *W. confusa* FS54 or FS54 B2 strains. The figure includes the different steps of the assay and the corresponding analyses conducted at the indicated incubation days (d). In addition, the CFU/mL of the inoculated samples were determined at time 0. The pH and the concentration of RF and metabolites present in the medium were also determined.

In recent years, there has been a growing interest in the food industry to enhance the RF levels in fermented foods as a natural alternative to vitamin supplementation. Despite this interest, until now, none of the commercially available plant-based beverages have been biofortified with vitamin B_2_ in situ by LAB fermentation. Therefore, with the aim of developing new functional fermented food beverages, the performance of the RF-overproducing strain FS54 B2 was evaluated alongside that of FS54 in three commercial plant-based beverages. The protocol design of the fermentations during 48 h at 30 °C and further storage for 32 days at 4 °C, as well as the sampling, is depicted in Figure 5.

**Figure 6:**
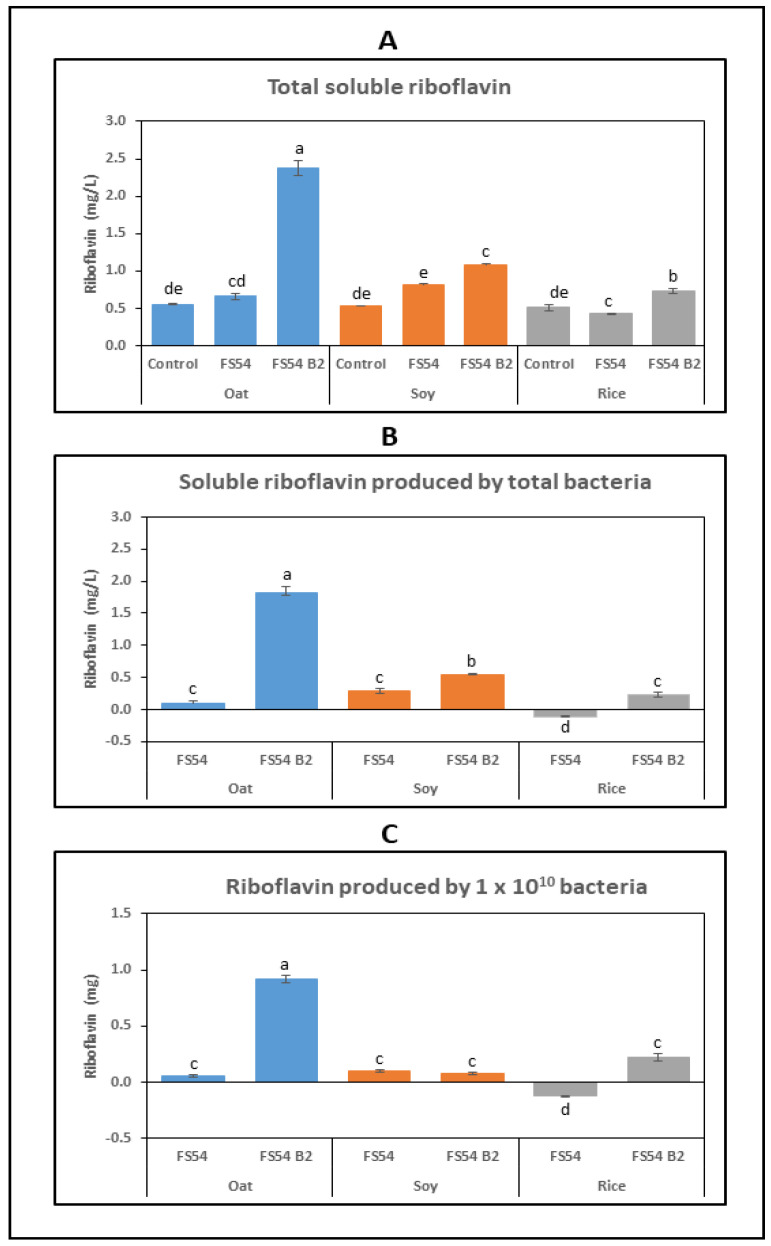
Detection of RF production in plant-based beverages fermented with FS54 or FS54 B2 strain. Commercial oat-, soy- and rice-based drinks were inoculated with either of the *W. confusa* strains (FS54 or FS54 B2) or not inoculated (control), and all were incubated 48 h at 30 °C prior to analysis. Concentrations of total soluble RF (**A**), RF produced by total bacteria (**B**) and RF produced by 1 × 10^11^ *Weissella* (**C**) are depicted. The different letters indicate statistically significant difference with one-way ANOVA analysis.

**Figure 7:**
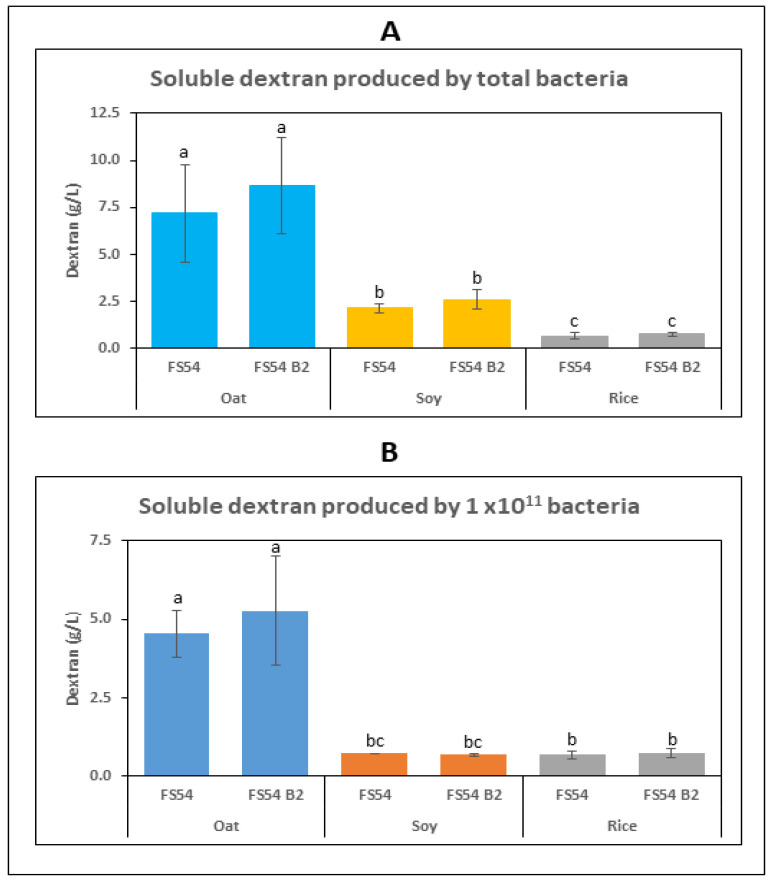
Detection of dextran production in plant-based beverages fermented with FS54 or FS54 B2 strain. Commercial oat-, soy- and rice-based drinks were inoculated with either of the *W. confusa* strains (FS54 or FS54 B2) or not inoculated (control), and all were incubated 48 h at 30 °C prior to analysis. Concentrations of total soluble dextran (**A**) and dextran produced by 1 × 10^11^ *Weissella* (**B**) are depicted. No dextran production was detected in the control sample. The different letters indicate statistically significant difference with one-way ANOVA analysis.

**Figure 8:**
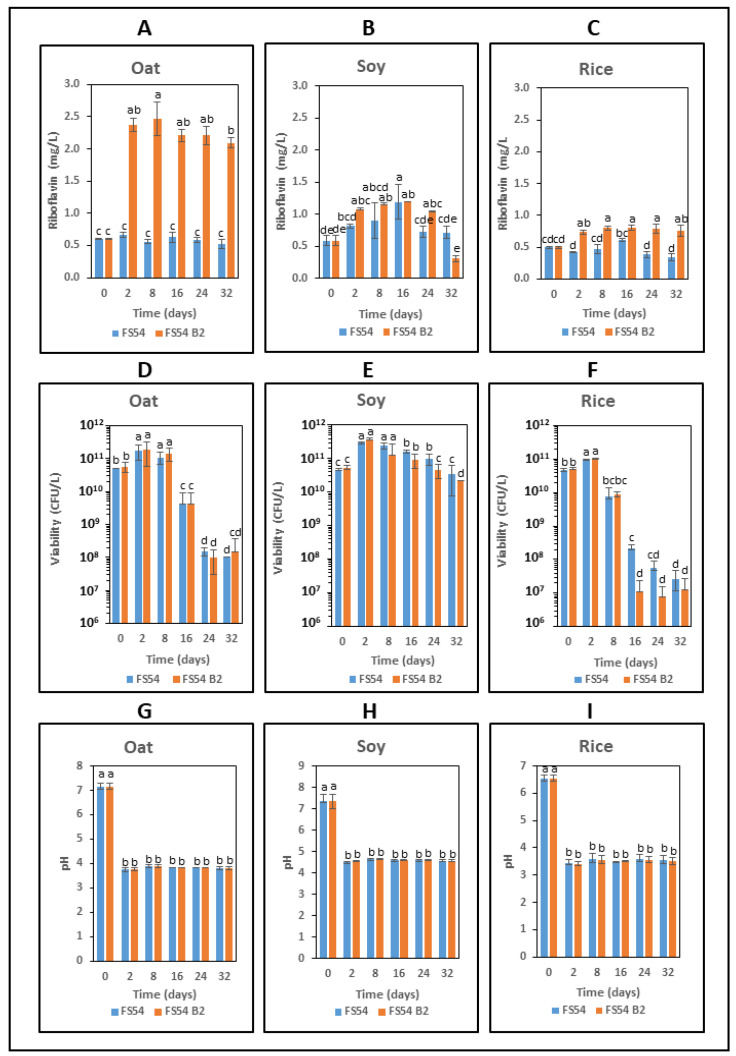
Analysis of oat- (**A**,**D**,**G**), soy- (**B**,**E**,**H**) and rice- (**C**,**F**,**I**) based beverages during fermentation and storage. The evolution of RF levels (**A**–**C**), bacterial viability (**D**–**F**) and pH (**G**–**I**) prior to or after 2 days of fermentation at 30 °C and during storage at 4 °C for over one month was determined. The different letters indicate statistically significant difference with one-way ANOVA analysis.

The oat-, soy- and rice-based beverages were supplemented with 5% sucrose to provide a substrate for the Dsr. Then, three fermentations were performed with each matrix: one control without inoculation, and the other two inoculated with either FS54 B2 or FS54 strain. After fermentation, the concentration of the RF (Figure 6) and dextran (Figure 7) as well the metabolic fluxes of the sugars in the inoculated beverages were assessed (Table 2). In addition, the pH-lowering capacity and the cell viability of the LAB were analyzed after fermentation (Figure 8). Finally, the evolution of the RF, the pH and the CFU/mL during the storage period was monitored (Figure 8).

#### 3.4.1. Detection of RF in the Beverages

After 2 days of fermentation, no visual detection of RF production by the *W. confusa* strains was observed in the brown-colored oat- and soy-based beverages. However, in the white-colored rice-based drinks, a pale yellow color and a more noticeable yellow color were observed after fermentation with the FS54 and FS54 B2 strains, respectively (Supplementary Figure S2).

Determination of the total soluble RF concentration in the fermented drinks (Figure 6A) showed significantly higher levels of the vitamin in the oat-, soy- and rice-matrices supplemented with FS54 B2, compared to the control and those treated with FS54. Particularly, in the oat matrix, the RF arose from 0.66 mg/L to 2.4 mg/L.

To further assess the influence of the *W. confusa* strains on the RF levels of the bacterial fermented drinks, and since the non-inoculated control beverages could contain indigenous microbiota, the RF values detected in these beverages were considered as background and subtracted from the values detected in the LAB-inoculated fermentations. The results are presented in Figure 6B, and they reveal that in oat and soy matrices, even the RF-producer FS54 strain biofortified the drink with 0.1 mg/L and 0.2 mg/L, respectively. In these two matrices, the RF overproducer FS54 B2 synthesized 1.82 mg/L and 0.5 mg/L, respectively. Moreover, in rice-based drinks, FS54 B2 produced only 0.3 mg/L and FS54 consumed the RF present in the matrix. Finally, when the values ascribed to the *W. confusa* strains were compared to the levels of LAB detected in the fermented drinks (Figure 6C), it was clear that, only in the oat and rice matrices, FS54 B2 was differentiated from FS54. In conclusion, the results presented indicate that only in the oat-based drink, FS54 B2 behaved as an efficient RF overproducer. In the rice matrix, the mutant differentiated from the wild-type strain (which reduces the initial RF levels) by increasing the RF concentration up to 0.3 mg/L. Finally, in the soy matrix, the FS54 B2 strain increased the RF concentration (0.5 mg/L production), but its behavior was not significantly different from that of FS54.

#### 3.4.2. Detection of Dextran in the Beverages

We previously established a method to determine the dextran concentration in beverages [45] by GC-MS detection and quantification of the isomaltose generated by the digestion of the polymer with the *Chaetomium erraticum* dextranase. Thus, using this method, we detected dextran production during the 2-day fermentation of the three matrices with either of the two *W. confusa* strains (Figure 7). Determination of the total soluble dextran concentration in the fermented drinks revealed a high production in the oat-based drinks of 8.7 g/L or 7.2 by the action of FS54 B2 or FS54, respectively (Figure 7A). Furthermore, the levels of dextran biofortification by FS54 or FS54 B2 were 2.1 g/L or 2.6 g/L in the soy-based drink and 0.6 g/L or 0.8 g/L in the rice-based drink. These differences in the biofortification of the three types of drinks were not ascribable to different levels of biomass, because they persisted when the values ascribed to the *W. confusa* strains were compared to the levels of LAB detected in the fermented drinks (Figure 7B). In conclusion, the results presented indicate that both strains have similar capacity to produce dextran in each of the three matrices tested and that the oat-based drink is the best environment for the Dsr synthetic activity involved in the polymer production.

#### 3.4.3. Analysis of Evolution of the RF, the pH and the Cell Viability During Fermentation and Storage of the Drinks

When the evolution of the total RF levels was analyzed from time 0, after fermentation at 30 °C and during the 30 days of further storage at 4 °C, the following results were obtained (Figure 8A–C).

In the oat matrix (Figure 8A), the RF levels obtained increased significantly upon fermentation (2 days versus time 0) and remained constant during the storage. In the case of FS54-inoculated drinks, no significant increment in vitamin B_2_ was observed after any treatment.

In the soy matrix (Figure 8B), the same behavior was observed in drinks inoculated with either *W. confusa* strain. Initially, a small gradual increase in RF levels was detected from time 0 to 16 days of treatment (2 days of fermentation and 14 days of storage). Afterwards, a slow sequential decrease in the RF concentration was observed until the end of the evaluated storage.

In the rice matrix (Figure 8C), as observed above (Figure 6), only FS54 B2 fermentation resulted in a slight increase in the RF levels, which remained basically constant throughout the storage period.

With regard to the bacterial viability during the experiment (Figure 8D–F), both LAB showed the same behavior in the three types of beverages. From initial values of approximately 5 × 10^10^ viable bacteria (CFU) per liter during fermentation in oat-, soy- and rice-based beverages, the levels increased to about 3 × 10^11^ CFU/L, 3 × 10^11^ CFU/L and 1 × 10^11^ CFU/L, respectively. Then, in rice drinks, the CFU/L started to decrease during the first week of storage, while this phenomenon was delayed in oat and soy drinks and was detected after 14 days of storage. Moreover, the profile of viability lost was different in the different types of beverages, when detected at the end of the experiment (one month of storage): the more drastic decrease was detected in rice drinks, the lowest in soy drinks, and an intermediate effect was observed in oat drinks, corresponding approximately to a final CFU/L of 2 × 10^7^, 1 × 10^10^ and 1 × 10^8^ CFU/L, respectively.

A low pH is a prerequisite to ensure the safety and microbiological stability of the beverage and to extend its shelf life. Thus, the fermentative efficacy of FS54 and FS54 B2 in the three plant-based drinks was tested by analyzing their ability to lower the pH (Figure 8G–I). Both LAB drastically reduced the pH from a neutral level (ranging from 7.2 to 6.5) in non-fermented beverages down to values as low 3.8, 4.8 and 3.4 in oat-, soy- and rice-based drinks, respectively. Furthermore, this characteristic remained unchanged throughout the storage period. Therefore, the overall results support the use of FS54 B2 for in situ biofortification of oat-based drink with RF, also providing the beverage with a potential beneficial increase in its shelf life accompanied by a significant bacterial load of *Weissella* with potential probiotic properties.

### 3.5. Metabolic Profiling of Plant-Based Beverages After Fermentation with the W. confusa FS54 and FS54 B2 Strains

To further investigate a potentially beneficial effect in the plant-based drinks, the levels of some sugars and their metabolites were determined after fermentation by a GC-MS analysis (Table 2).

The Dsr catalyzes the synthesis of dextran by hydrolysis of the sucrose disaccharide, producing fructose, free glucose and dextran by elongation of the polymer composed of glucopyranose molecules. Furthermore, we have previously shown that the *W. confusa* FS54 strain is capable of producing mannitol, presumably from fructose in a reaction catalyzed by the mannitol dehydrogenase [16]. Thus, the addition of fructose plus mannitol production will indicate the efficiency of the *W. confusa* Dsr in hydrolyze sucrose in the different plant-based drinks. Most of the initial 182.9 mM sucrose was consumed in the FS54 and FS54 B2 fermentations in the oat-based beverage, with a yield of 138.1 mM and of 178.1 mM of fructose plus mannitol, respectively. Thus, the *W. confusa* Dsr was very efficient in this matrix. In the soy-based drink, a similar situation took place; no detectable levels of sucrose were observed in the beverages fermented with either FS54 or FS54 B2, and the consumption of 183.1 mM sucrose yielded 131.4 mM and 139.3 mM fructose plus mannitol in the drinks supplemented with either the wild-type or the mutant strain, respectively. Finally, from the initial level of 141.7 mM sucrose in rice-based drinks, still high concentrations of this disaccharide (40.4 mM and 53.8 mM) were detected in fermentations with either FS54 or FS54 B2. In addition, according to the fructose plus mannitol levels detected, only 21.8 mM and 16.6 mM were consumed by the Dsr of the wild-type and mutant strains, respectively. These results indicated a similar hydrolytic activity by Dsr in oat and soy matrices, approximately 3-fold higher than in the rice matrix.

The drinks used in this work were subjected to ultra-high temperature (UHT) treatment prior to commercialization. Therefore, we would like to state that it is known that some bacteria that are able to sporulate, such as very-high-heat-resistant mesophilic species of *Bacillus*, have been isolated from UHT-treated drinks [50]. Therefore, heat-resistant bacteria present in the drink should be responsible for the consumption of sucrose, which correlated with the low levels of disaccharide detected in the control sample (38.6 mM) after 2 days of fermentation. It is noteworthy that no bacteria could be detected in MRSG when the fermented control samples were plated. No LAB were detected in the control fermented drink, and consequently, these type of bacteria appeared not to be responsible for consuming sucrose.

Likewise, maltose was present in oat- and rice-based beverages with values ranging from 43 to 49 mM in the non-inoculated beverage and from 43 to 54 mM in the bacterial fermented drinks, with no statistically significant differences between them.

As expected from the decrease in the pH detected after fermentation (Figure 8G–I), only lactic acid was detected in all the plant-based beverages inoculated with both *Weissella* strains. The levels detected were not very high, around 10 mM in oat- and soy-based drinks and around 1 mM in the rice-based beverage. However, it should be noted that *W. confusa* is a heterofermentative microorganism, and presumably, other acidic compounds, such as acetic acid, which were not detected by the GC-MS analysis under our conditions, were present in the fermented drinks.

The metabolic analysis also showed that fermentation of oat-based beverages with either FS54 or FS54 B2 strains resulted in the production of oligosaccharides with potential prebiotic properties. Both strains displayed similar metabolic profiles, but FS54 B2 produced slightly higher levels of isomaltose (2.2 mM), isomaltotriose (0.9 mM), and panose (10.2 mM). It is also important to note that the commercial unfermented oat-based drink showed a level of 15.6 mM of maltotriosa, which was slightly increased to 23 mM in the FS54 B2-fermented drinks, although this difference was not statistically significant. Furthermore, low levels of panose (around 3 mM) were detected in the rice-based drinks when fermented with either LAB. In conclusion, the above results indicate that *W. confusa* FS54 B2 has the potential to produce functional fermented oat drinks, enriched with RF, prebiotic oligosaccharides and mannitol, a polyol with antioxidant properties [51]. The strain’s ability to produce vitamin B_2_ and dextran, along with its stable performance during fermentation and storage, supports its application in biofortifying plant-based beverages.

## 4. Discussion

In this study, we successfully isolated, identified and initially characterized the FS54 B2 strain, which, to our knowledge, is the first RF-overproducing, dextran-producing *W. confusa* strain. The FS54 B2 strain was derived from the parental strain FS54 by the method developed by Burgess et al. [31] to isolate RF-overproducing LAB mutants by treatment with roseoflavin, with the modifications previously made by us to isolate *W. cibaria* mutants [35].

*W. confusa* FS54 belonged to the laboratory collection of microorganisms and active biomolecules (LMBA) within the Faculty of Sciences Tunis. It was originally isolated from olive tree rhizosphere in Tunisia, with the aim of diversifying potential probiotic LAB sources [42]. The strain was subsequently identified as a robust exopolysaccharide producer, and the polymer synthesized by the bacteria was purified and structurally characterized as dextran with a high molecular weight of 6 × 10^7^ Da [16]. These types of biopolymers of lactobacilli and *Leuconostoc* strains have immunomodulatory properties [12].

The present work confirmed the strong capacity of *W. confusa* FS54 to produce dextran in RAMS medium up to 21.1 mM at OD_600nm_ of 3.1 (Table 1), consistent with our previous findings when the bacterium produced 0.8 g/L at OD_600nm_ of 1.6 upon growth in CDMS (a chemically defined medium) [16]. Moreover, here, we have detected the same production levels for both the FS54 and the FS54 B2 strains, indicating no effect on dextran production in the mutant strain.

In terms of the RF production, in one of our previous works, we isolated several roseoflavin-resistant *W. cibaria* mutants that, when grown in RAMS lacking RF, synthetized the vitamin at levels ranging from 0.8 mg/L to 6.5 mg/L, with BAL3C-5 C120T being the best RF-overproducing mutant [45]. In this study, *W. confusa* FS54 B2 produced 4.9 mg/L of RF, when grown in RAMS under microaerophilic conditions (45 mL without agitation and in a 50 mL screw-cap bottle) (Table 1). This level of RF synthesis in RAMS medium increased up to 5.9 mg/mL (Figure 3) when the strain was grown under aerobic conditions in microtiter plates (200 μL in microtiter plates with agitation every 30 min). Thus, in the absence of RF, *W. confusa* FS54 B2 synthesized vitamin B_2_ at levels close to those of the *W. cibaria* BAL3C-5 C120T, the best mutant of this species described to date.

When compared to other RF-overproducing LAB mutants isolated and obtained through roseoflavin treatment, FS54 B2 exhibited higher synthesis than most reported lactobacilli (0.6–3.7 mg/L) [31,32,38,52,53,54], *L. lactis* (0.9 mg/L) [30], and *L. mesenteroides* [(0.6 mg/L) [10]. Although two LAB, *L. plantarum* HY7715 (14.5 mg/L) [55] and the spontaneous natural RF overproducer *L. reuteri* AMBV339 (18.2 mg/L) [31] strain, have been reported to produce higher levels of the vitamin B_2_, these LAB were grown at significantly higher biomass levels, with 10- and 60-fold higher biomass than *W. cibaria* BAL3C-5 C120T [45] and *W. confusa* FS54 B2. Thus, FS54 B2 showed promising RF overproduction compared to other LAB.

Moreover, in RAMS and RAMG, FS54 B2 released by diffusion or secretion more than 84% of the RF produced in the cytoplasm (Table 1), a good property for the food matrix’s enrichment with the vitamin by fermentation. Therefore, FS54 B2 deserves to be evaluated for its capability for in situ biofortification of food and/or drinks with the aim of developing new functional foods.

The growing consumer preference for natural products and clean labels is particularly noteworthy. LAB is therefore emerging as a valuable tool for improving the functionality and nutritional content of foods. At the same time, there has been a significant increase in the availability of novel plant-based foods and beverages, driven by the growing demand for alternatives to animal products. This trend further highlights the significance of innovations such as LAB-mediated biofortification in meeting evolving consumer preferences and nutritional needs [56].

Therefore, in this study, we have used FS54 B2 to ferment commercial plant beverages based on oat, soy, and rice matrices, as they are currently very popular among the general population and of particular interest to individuals with lactose intolerance and allergies, as well as the vegan population. Fermentation with FS54 B2 was able to enrich the three drinks with the RF (Figure 6) at different final levels: the oat-based drink up to 2.4 mg/L, the soy-based drink up to 1.1 mg/L and the rice-based drink up to 0.7 mg/L (Figure 6).

These results are in accordance with previous research, which has consistently shown that RF overproducer LAB mutants can significantly enhance the vitamin B_2_ production in fermented foods. In this context, recent research has underscored the efficacy of various LAB strains in increasing the RF levels in foods through biofortification. For example, *L. fermentum* and *W. cibaria* strains have been shown to be fortified with RF concentrations of 6.6 µg/g and 3.5 µg/g, respectively [34,35,40]. Similarly, *L. plantarum* strains increased the RF concentration in soy beverages to 0.7–1.9 µg/mL [53], while the RF levels in kefir-like beverages ranged from 0.5 to 1.5 mg/L depending on the fermentation conditions [38]. We have also recently shown, using the same type of commercial beverages employed in this study, that the *W. cibaria* BAL3C-5 C120T, like *W. confusa* FS54 B2, has the best performance in oat-based beverages, increasing the RF levels up to 2.7 mg/L [45], a value only slightly higher than that obtained when fermenting with the *W. confusa* mutant (Figure 6).

Plant-based beverages are intended for consumption after refrigerated storage, similar to dairy products. In this context, both LAB retained the vitamin B_2_ levels during storage at 4 °C for one month after fermentation (Figure 7 and [45]), and both decreased the pH to about 3.8 during fermentation. These properties are crucial requirements to guarantee the safety, the microbiological stability and the functionality of the beverage, as well as to extend its shelf life.

Given the European Food Safety Authority (EFSA)-recommended daily intake values for RF (0.4 to 2 mg/day depending on demographic factors), the results obtained here highlight the practical benefits of the RF biofortification of oat beverages with *W. confusa* FS54 B2. For instance, a 200 mL serving of the biofortified oat beverage analyzed in this study will provide approximately 0.48 mg of RF per day. This contribution is significant in the context of a balanced diet and can help the consumer to meet a significant portion of the daily RF requirement. These estimates highlight the potential efficacy of the *W. confusa* FS54 B2 strain in improving the nutritional quality of food products and suggest that the inclusion of such biofortified beverages could be a valuable strategy for meeting dietary RF needs.

Furthermore, the metabolic analysis of the oat-based fermented drinks performed here revealed that FS54 B2 biofortified them with 10.2 mM panose, 2.2 mM isomaltose and 0.9 mM isomaltotriose oligosaccharides (Table 2). This fortification should contribute to the functionality of the oat beverage, as these oligosaccharides provide several health benefits, including resistance to digestive enzymes in the gastrointestinal tract, which leads to a low caloric value and glycemic index. They also support the growth of probiotic bacteria, which, in turn, produce beneficial short-chain fatty acids, without fostering the growth of pathogenic bacteria. In addition, these oligosaccharides maintain excellent stability under typical food processing conditions such as low pH and moderately high temperatures [57].

Interestingly, FS54 B2 produced significantly higher levels of panose compared to *W. cibaria* BAL3C-5 C120T (10.2 mM versus 0.3 mM) [45]. This difference could be connected with the dextranase activity of the *W. confusa* strain. This hypothesis is based on the following facts. We have previously found, in a time course experiment, that the concentration of dextran synthesized by the parental FS54 strain decreased during incubation [16]. Moreover, we have shown here that FS54 B2 like its parental possesses dextranase activity (Figure 2B), and we have not observed this activity for the *W. cibaria* BAL3C-5 C120T strain (unpublished results), Consequently, the high production of panose by FS54 B2 could be due to its dextranase activity, which could hydrolyze partially the synthesized dextran present in the oat-based drink.

Moreover, FS54 B2 synthesized and released high levels of mannitol (87 mM) into the beverage (Table 2), compared to no detectable production by BAL3C-5 C120T [45]. This polyol has antioxidant activity, providing protection against oxidative damage by oxygen radicals, and is a non-metabolizable sweetener [51]. Therefore, its presence should provide increased functionality to the fermented oat drink.

In addition, FS54 B2 was able to produce in the oat-based drink (Figure 7) considerably higher levels of dextran than BAL3C-5 C120T (48 mM versus 11 mM [45]). This fact is important for its potential impact on the rheological properties of the fermented beverages, and also because dextran, with postbiotic benefits for human health [18,19], will contribute to increase the functionality of the oat-based drinks.

In conclusion, this study demonstrates the potential of *W. confusa* FS54 B2 for in situ biofortification of oat-based beverages, presenting a promising approach for developing novel functional drinks. Further characterization of the strain concerning its probiotic properties, in vitro and in vivo, and optimization of oat-based fermentation conditions, as well as testing of other food matrices, will be essential for maximizing the strain’s utility in food fortification.

## 5. Patents

Part of this research has been included in the Tunisian patent application N° TN 2024/0217.

## Figures and Tables

**Table 1:** Analysis of the RF and the dextran production by the *W. confusa* FS54 and FS54 B2 strains.

*W. confusa* Strain	Medium	Total RF ^1^ (mg/L)	Free RF ^2^ (mg/L)	Free RF Total RF (%)	OD_600nm_	Dextran ^3^ (g/L)
**FS54 B2**	RAMS	4.9 ± 0.7 ^a^	4.7 ± 0.2 ^a^	95.9	3.1 ± 0.3 ^b^	3.8 ± 0.1 ^a^
	RAMG	3.9 ± 0.3 ^b^	3.3 ± 0.2 ^b^	84.6	4.3 ± 0.2 ^a^	ND
**FS54**	RAMS	0.3 ± 0.0 ^c^	0.2 ± 0.0 ^c^	66.6	4.01 ± 0.2 ^a^	3.8 ± 0.1 ^a^
	RAMG	0.2 ± 0.0 ^c^	0.1 ± 0.0 ^c^	50.0	4.0 ± 0.1 ^a^	ND

Bacteria were grown in either RAMG or RAMS during 16 h at 30 °C prior to analysis. The different letters indicate statistically significant differences with one-way ANOVA analysis. ^1^ Total RF: the vitamin B_2_ levels were determined using samples of the cultures. ^2^ Free RF: the vitamin B_2_ levels were determined on samples of culture supernatants. ^3^ Dextran concentration was determined in the culture supernatants. ND: undetectable levels.

**Table 2:** Metabolic analysis of the plant-based beverages after fermentation with *W. confusa* FS54 and FS54 B2 strains.

Fermented Drink	Sucrose (mM)	Fructose (mM)	Mannitol (mM)	Glucose (mM)	Lactic Acid (mM)	Maltose (mM)	Isomaltose (mM)	Maltotriose (mM)	Isomaltotriose (mM)	Panose (mM)
**Oat** **Control**	43.4 ± 8.0 ^b^	4.4 ± 0.9 ^b^	ND	60.8 ± 12.1 ^a^	ND	43.1 ± 6.4 ^a^	ND	15.6 ± 1.9 ^a^	ND	ND
**FS54**	1.9 ± 0. 1^a^	72.7 ± 0.1 ^a^	69.8 ± 1.8 ^a^	74.9 ± 2.4 ^a^	7.7 ± 0.6 ^a^	43.2 ±1.2 ^a^	1.6 ± 0.1 ^b^	17.6 ± 6.0 ^a^	0.7 ± 0.1 ^b^	8.1 ± 0.8 ^a^
**FS54 B2**	1.4 ± 0.7 ^a^	89.8 ± 1.1 ^a^	87.0 ± 7.7 ^a^	92.1 ± 7.3 ^a^	8.7 ± 0.7 ^a^	54.1 ± 5.5 ^a^	2.2 ± 0.0 ^a^	22.3 ± 9.7 ^a^	0.9 ± 0.1 ^a^	10.2 ± 0.5 ^a^
**Soy** **Control**	61.6 ± 0.7 ^a^	1.6 ±0.1 ^b^	ND	0.9 ± 0.1 ^b^	ND	0.42 ± 0.1 ^b^	ND	ND	ND	ND
**FS54**	ND	88.1 ± 25.9 ^a^	44.9 ± 7.5 ^a^	32.3 ± 4.7 ^a^	11.2 ± 1.3 ^a^	1.27 ± 0.2 ^ab^	ND	ND	ND	ND
**FS54 B2**	ND	95.6 ± 15.5 ^a^	48.3 ± 3.3 ^a^	40.1 ± 6.4 ^a^	12.5 ± 0.7 ^a^	1.06 ± 0.2 ^a^	ND	ND	ND	ND
**Rice** **Control**	38.6 ± 5.0 ^a^	9.7 ± 6.8 ^a^	ND	67.9± 14.5 ^a^	ND	48.8 ± 9.0 ^a^	ND	35.2 ± 4.4 ^a^	ND	ND
**FS54**	40.3 ± 13.5 ^a^	16.2 ± 10.0 ^a^	5.6 ± 2.5 ^a^	67.7 ± 3.4 ^a^	0.8 ± 0.0 ^ab^	42.1 ± 11.9 ^a^	ND	31.0 ± 5.4 ^a^	ND	2.8 ± 0.2 ^a^
**FS54 B2**	55.1 ± 3.4 ^a^	9.6 ± 1.4 ^a^	7.0 ± 3.1 ^a^	78.1 ± 1.0 ^a^	1.6 ± 0.4 ^a^	53.8 ± 1.9 ^a^	ND	34.2 ± 2.4 ^a^	ND	3.2 ± 0.8 ^a^

Oat-based (oat), soy-based (soy), and rice-based (rice) drinks supplemented with sucrose and inoculated with either *W. confusa* strain (FS54) or (FS54 B2) or without addition of bacteria (control) were fermented for 48 h. The different letters indicate statistically significant difference with one-way ANOVA analysis. The initial concentration of sucrose in the commercial beverages supplemented with this disaccharide was 182.9 mM in oat-based, 183.1 mM in soy-based and 141.7 mM in rice-based drinks. ND: undetectable levels.

## Data Availability

The original contributions presented in the study are included in the article and Supplementary Material, further inquiries can be directed to the corresponding author.

## Supplementary Materials

The following supporting information can be downloaded at: https://www.mdpi.com/article/10.3390/foods13244112/s1, Supplementary Figure S1. Riboflavin calibration curves. Riboflavin solutions were prepared at 10 mg/mL and sequentially diluted in: Riboflavina Assay Medium (RAM) (A), oat-based drink (B), soy-based drink (C) and rice-based drink (D). Then aliquots of 200 μL of the solutions were dispensed in 96-well polystyrene optical bottom plate (Thermo Fisher Scientific), and the riboflavin fluorescence was measured using a Varioskan Flask System (Thermo Fisher Scientific). Supplementary Figure S2. Photographs showing fermented plant beverages. Commercial oat-, soy- and rice-based drinks were inoculated with either of the W. confusa strains (FS54 or FS54 B2) or not inoculated (control) and all were incubated 48 hours at 30 °C.
