# Supplementary material for: Riboflavin- and Dextran-Producing Weissella confusa FS54 B2: Characterization and Testing for Development of Fermented Plant-Based Beverages
Source: Foods. 2024 Dec 19;13(24):4112. doi: 10.3390/foods13244112 (PMC11675806; doi:10.3390/foods13244112)
Supplement: Supplementary file 1 [file foods-13-04112-s001.zip › foods-3285091-supplementary.pdf]

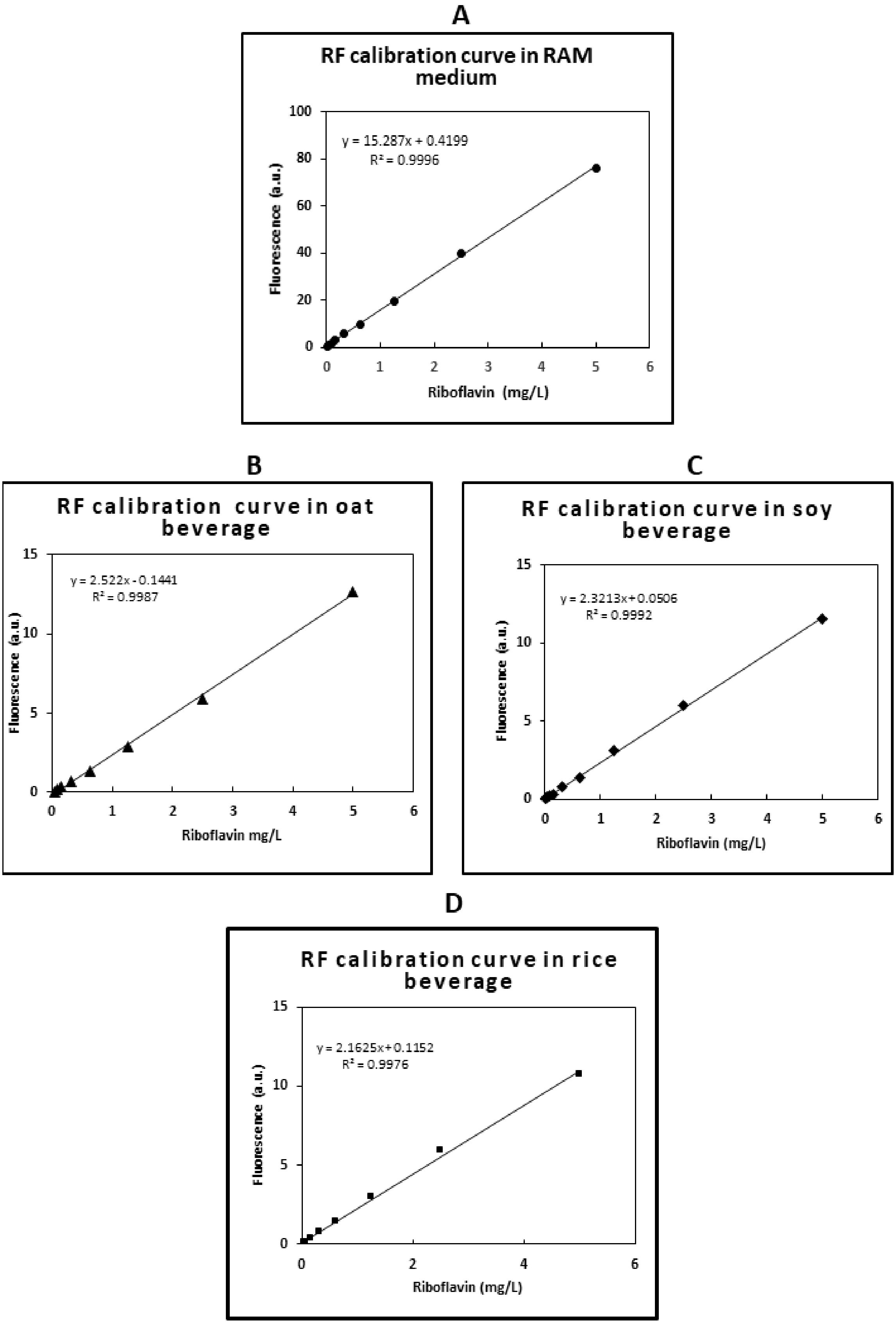

Supplementary Figure S1. Riboflavin calibration curves. Riboflavin solutions were prepared at 10 mg/mL and sequentially diluted in: Riboflavina Assay Medium (RAM) **(A),** oat-based drink **(B),** soy-based drink **(C)** and rice-based drink **(D)**. Then aliquots of 200 μL of the solutions were dispensed in 96-well polystyrene optical bottom plate (Thermo Fisher Scientific), and the riboflavin fluorescence was measured using a Varioskan Flask System (Thermo Fisher Scientific).

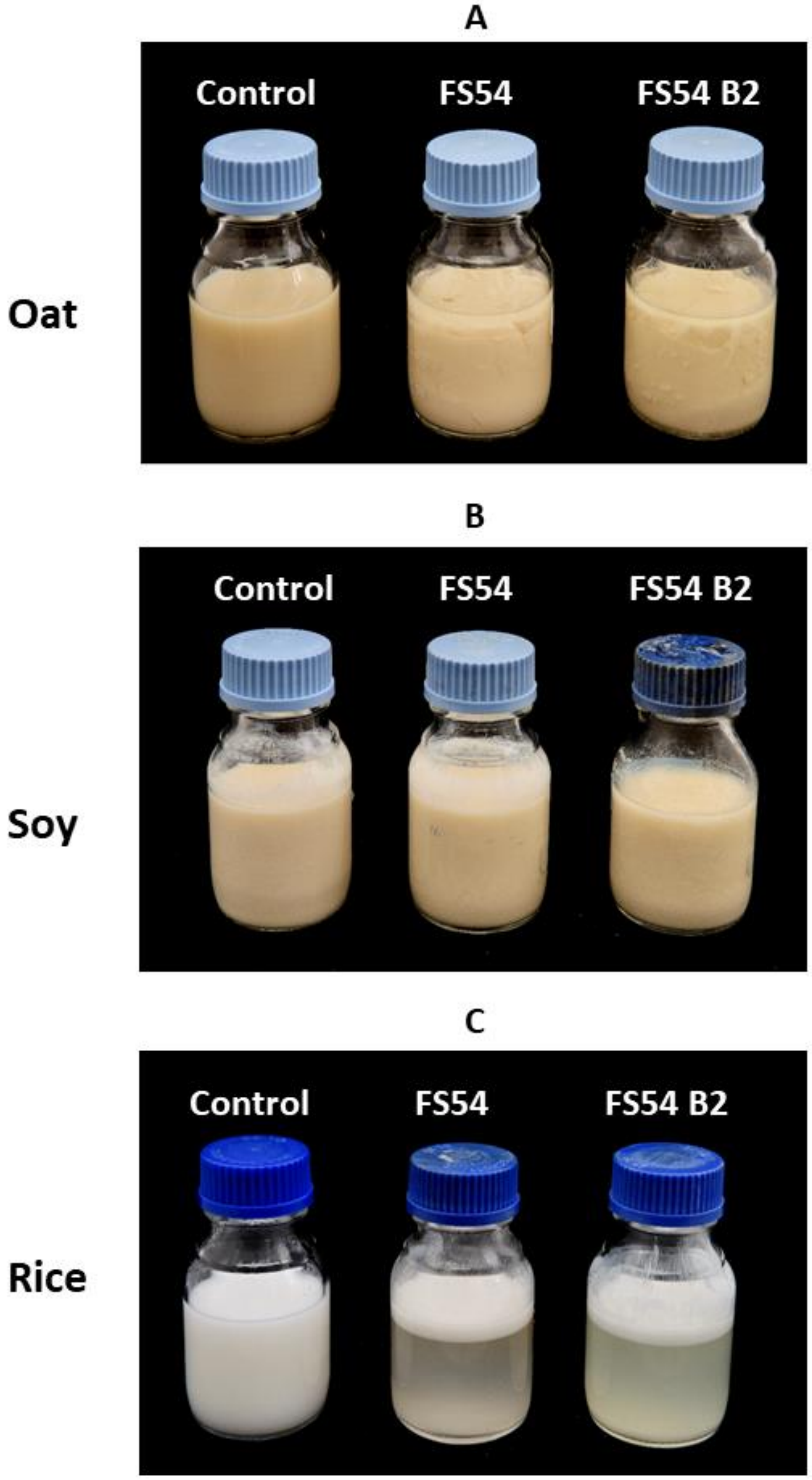

Supplementary Figure S2. Photographs showing fermented plant beverages. Commercial oat-, soy- and rice-based drinks were inoculated with either of the *W. confusa* strains (FS54 or FS54 B2) or not inoculated (control) and all were incubated 48 hours at 30 °C.
